# ZIF67-ZIF8@MFC-Derived Co-Zn/NC Interconnected Frameworks Combined with Perfluorosulfonic Acid Polymer as a Highly Efficient and Stable Composite Electrocatalyst for Oxygen Reduction Reactions

**DOI:** 10.3390/polym16040505

**Published:** 2024-02-12

**Authors:** Hongjie Meng, Jingnan Song, Yongming Zhang

**Affiliations:** School of Chemistry and Chemical Engineering, Center of Hydrogen Science, Shanghai Jiao Tong University, Shanghai 200240, China; menghongjie@sjtu.edu.cn

**Keywords:** ZIF67-ZIF8, Co/N-codoped polymer composite catalyst, carbon nanofibers, graphitic carbon, oxygen reduction reaction, catalytic stability

## Abstract

The development of precious metal-free (M-N-C) catalysts for the oxygen reduction reaction (ORR) is considered crucial for reducing fuel cell costs. Herein, Co-Zn/NC interconnected frameworks with uniformly dispersed Co nanoparticles and graphitic carbon are designed and successfully synthesized through the in situ growth of zeolitic imidazolate frameworks (ZIF67 and ZIF8) along with biomass nano-microfibrillar cellulose (MFC), followed by pyrolysis. A Co-Zn/NC composite is prepared by combining Co-Zn/NC with a perfluorosulfonic acid polymer. The Co-Zn/NC composite catalyst exhibits excellent ORR catalytic activity (E_0_ = 0.974 V vs. RHE, E_1/2_ = 0.858 V vs. RHE) and good long-term durability, with 90% current retention after 10000s, surpassing that of commercial Pt/C in alkaline media. The hierarchical porous structure, coupled with the uniform distribution of Co nanoparticles and nitrogen doping, contributes to superior electrocatalytic performance, while the interconnected frameworks and graphitic carbon ensure good stability. Additionally, the Co-Zn/NC composite demonstrates promising applications in acidic media. This strategy offers significant guidance to develop advanced non-precious metal carbon-based catalysts for highly efficient and stable ORR.

## 1. Introduction 

The urgent need to develop novel sustainable and clean energy arises from the global energy crisis and environmental pollution stemming from traditional fossil fuels. Fuel cells (FCs) are environmentally friendly and energy-efficient devices that can directly convert chemical energy into electrical energy [1,2,3]. Because of the slow kinetic process of the cathode oxygen reduction reaction (ORR) in fuel cells, the electrocatalysts used for cathode ORR have attracted much attention [4,5]. The precious metal Pt-based catalysts, although currently the most effective and widely utilized for ORR, encounter challenges in large-scale commercial applications due to limited resource reserves, high costs, and poor long-term durability (susceptibility to carbon monoxide/methanol poisoning) [6,7,8]. Therefore, it is urgent to develop a cost-effective, stable, and efficient non-precious metal electrocatalyst for high-performance ORR [9,10].

In recent years, various nitrogen-doped carbon (N-C) [11] or metal-nitrogen-doped carbon (M-N-C, M=Fe, Co, Ni, Mn, etc.) [12] catalysts synthesized through extensive research have been recognized as exceptional ORR electrocatalyst candidates in both acidic and alkaline electrolytes. Notably, as a novel high-activity catalyst for ORR, M-N-C electrocatalysts reported in some studies have demonstrated ORR catalytic performance comparable to or even surpassing that of commercial Pt/C catalysts [13,14,15]. Liang group prepared the controllable construction of core-shell polymer@zeolitic imidazolate framework fiber (PAN@ZIFs) derived heteroatom-doped carbon nanofiber network (CNF@Zn/CoNC) through an in situ growth method followed by pyrolysis, which showed brilliant ORR catalytic activity and durability [16]. Mu et al. synthesized an advanced Co-N_x_/C nanorod array catalyst derived from 3D ZIF nanocrystals with superior electrocatalytic performance toward ORR compared to commercial Pt/C, which further displays the high performance of Zn-air batteries with high cycling stability [17]. Muhler et al. constructed core-shell Co@Co_3_O_4_ nanoparticles encapsulated in carbon nanotube-grafted N-doped carbon polyhedra by the pyrolysis of ZIF-67 in a reductive H_2_ atmosphere and subsequent controlled oxidative calcination. The as-prepared catalyst outperforms highly active oxygen electrodes, surpassing commercial Pt/C in alkaline media [18]. Li et al. developed a controllable hierarchically porous carbon nanofiber (Fe-NC@CBC) by in situ growth of controllable Fe-based organic frameworks on bacterial cellulose, which exhibits efficient electrocatalytic performance surpassing that of commercial Pt/C in microbial fuel cells [19]. Lin group prepared a cellulose/graphene oxide/ZIF8-derived highly conductivity integrated film electrode for a supercapacitor; the goal product carbonized-CNFs/GO/ZIF8 (CCGZ) exhibits high conductivity and the all-solid-state device achieves a desirable energy density [20]. Li et al. designed a cellulose nanofibers@zeolitic imidazolate framework-derived mesoporous carbon-supported nanoscale CoFe_2O4_/CoFe hybrid composition as a trifunctional electrocatalyst for Zn-air battery, which presents excellent power density and outstanding cycling stability due to the effective exposure nanoscale-dispersed active sites and abundant mesoporous structure [21]. Lee group developed a cellulose nanofiber composite with a bimetallic zeolite imidazole framework (CNF-HZNPC) for electrochemical supercapacitors. The CNF-HZNPC composite electrodes show superior electrochemical performance and cycling stability [22]. ZIF-derived materials or carbonized ZIFs have proven to be ideal candidates for ORR catalysts or supports due to their outstanding characteristics, including well-defined hollow structures with ultrahigh surface area, excellent electrical conductivity, high nitrogen content, and abundant M-N coordination. These enable the potential for designing and generating more active and durable catalysts [23,24,25].

Despite significant advancements that have been achieved in electrocatalytic activities, challenges still exist in long-term operational stability for ZIF-derived electrocatalysts. [26] The unavoidable structure collapse and aggregation of the ZIFs during carbonization usually damage their well-ordered porous morphology, thereby compromising their performance [27]. Furthermore, leaching of the metal active sites (caused by chemical/electrochemical dissolution or destruction of the carbon matrix) and carbon corrosion (chemical attack by H_2_O_2_/free radicals or electrochemical oxidation at high potential) are considered to be the primary factors in the degradation of M-N-C catalysts [28,29,30]. Incorporating carbon fiber to anchor ZIFs and increasing the graphitization degree in the carbon matrix are effective strategies to prevent or reduce the aggregation of ZIF derivatives and improve the dispersion of the catalytic active sites and stability [31,32,33].

Nano-microfibrillar cellulose (MFC) is very easy to disperse in alcohol and facile to carbonize into carbon nanofibers (CNFs) [34]. Transition metals can catalyze the formation of graphitization during pyrolysis and carbonization. [35] Herein, novel interconnected frameworks with uniformly dispersed Co nanoparticles and graphitic carbon (denoted as Co-Zn/NC) are designed and successfully developed through the in situ growth of zeolitic imidazolate frameworks (ZIF67 and ZIF8) within MFC-dispersed methanol solution, followed by pyrolysis. Where the integration of the Cobalt metal-organic framework (ZIF-67) and Zinc metal-organic framework (ZIF-8) enables the utilization of both microporous and macroporous structures while allowing for adjustment of the Co content. The transition metal Co can not only form efficient active sites for ORR but also serve as catalysts to facilitate graphitization. Co-Zn/NC composite catalysts are prepared by incorporating Co-Zn/NC with a perfluorosulfonic acid polymer. Consequently, Co-Zn/NC composite catalysts exhibit efficient electrocatalytic activities, good long-term stability, and excellent methanol resistance, surpassing that of commercial Pt/C in alkaline media, as well as promising potential for ORR in acidic media. The hierarchical porous structure coupled with the uniform distribution of Co/N-C active sites contributes to superior electrocatalytic performance, while the interconnected frameworks and graphitic carbon ensure good stability. This study offers novel insights for the design and development of Co-N-C ORR catalysts with exceptional catalytic activity and excellent long-term stability. 

## 2. Experimental Section

### 2.1. Materials and Chemicals 

Nano-microfibrillar cellulose (MFC), composed of (C_6_H_5_O_5_)n, was obtained from Hangzhou Tuomu Technology Co., Ltd., Hangzhou City, Zhejiang Province, China. MFC looks like a gel and can be dispersed in solvents, such as water, methanol, and ethanol. Its radial dimension is in the range of 20–60 nm, and the length is 2–30 μm. Other chemicals including methanol (AnalyticalReagent, >99.7%), ethanol (AnalyticalReagent, >99.5%), 2-methylimidazole, potassium hydroxide (>95%), sulfuric acid (>98%), cobalt (II) nitrate hexahydrate (Co(NO_3_)_2_·6H_2_O), and Zinc(II) nitrate hexahydrate (Zn(NO_3_)_2_·6H_2_O) were procured from Shanghai Maclin Biochemical Technology Co., Ltd. (Pudong, Shanghai, China) and used directly. Nafion^®^ solution with a concentration of 5 wt. % was acquired from DuPont Company (Wilmington, DE, USA). Deionized water was used throughout the experiment. 

### 2.2. Preparation of ZIF8@MFC, ZIF67@MFC, and ZIF67-ZIF8@MFC and Zn/NC, Co/NC, and Co-Zn/NC

Firstly, 0.730 g of Zinc(II) nitrate hexahydrate was dissolved in 50 mL of methanol and stirred for 30 min. The obtained dispersion solution was named solution A. 1.63 g of 2-methylimidazole (2-Ml) was added to 50 mL of methanol under continuous magnetic stirring for 30 min, named solution B. Secondly, MFC (1.0 g) was dispersed into the methanol solution of Zinc(II) nitrate (solution A) under continuous magnetic stirring for 30 min, and then mixed solution A with solution B (the 50 mL methanol solution of 2-methylimidazole). Finally, after magnetic stirring for 24 h at room temperature, the resulting precursor products were collected via centrifugation for 5 min with a centrifugation rate of 8000 rpm/min. It was then washed three times with methanol and freeze-dried overnight in a freeze-dryer. The resulting sample was named ZIF8@MFC. ZIF67@MFC was prepared through the same procedure with Zn(NO_3_)_2_·6H_2_O replaced by an equal molar amount (0.714 g) of Co(NO_3_)_2_·6H_2_O. The ZIF67-ZIF8@MFC composites were synthesized using the same procedure, with fixed contents of 2-methylimidazole (1.63 g) and MFC (1.0 g), while varying the molar ratio between Co(NO_3_)_2_·6H_2_O and Zn(NO_3_)_2_·6H_2_O. The ratio of Co(NO_3_)_2_·6H_2_O to the sum of Co(NO_3_)_2_·6H_2_O and Zn(NO_3_)_2_·6H_2_O is 10%, 20%, 30%, and 50%, respectively. The obtained samples were designated as ZIF67-ZIF8@MFC-X (X = 10%, 20%, 30%, 50%), with detailed sample compositions provided in Table S1. The prepared precursor samples ZIF8@MFC, ZIF67@MFC, and ZIF67-ZIF8@MFC-X (X = 10%, 20%, 30%, 50%) were pyrolyzed at 800 °C in a nitrogen atmosphere for 2 h with a heating rate of 5 °C/min, and the final products were designated as Zn/NC, Co/NC, and Co-Zn/NC−10%, −20%, −30%, and −50%, respectively. ZIF67@MFC precursors calcinated at 600 °C, 700 °C, and 800 °C under identical calcination conditions were named Co/NC-T (T = 600, 700, and 800).

### 2.3. Preparation of Zn/NC, Co/NC, and Co-Zn/NC Composites 

The composite catalyst ink was prepared by dispersing 1 mg catalyst (Zn/NC, Co/NC, and Co-Zn/NC) into the mixed solution of 10 μL perfluorosulfonic acid polymer solution (5 wt.% Nafion 117 solution) and 200 μL hydroalcoholic solution (V_water_:V_alcohol_ = 4:1) under ultrasonic homogenization. Then the composite catalyst ink was dripped on the glassy carbon rotating disk electrode with a catalyst loading of about 0.3 mg/cm^2^. After being dried with the infrared lamp, a coating of the composite catalyst is formed on the glass carbon electrode. 

### 2.4. Physical Characterization

The morphological microstructures of the prepared samples were observed by SEM (Nova NanoSEM 450, FEI Company, Oregon, USA) at 15 kV in a high vacuum and TEM (Talos F200X, Thermo Scientific, Waltham, MA, USA), respectively. The structure and composition of the prepared catalysts were characterized by XRD on a Cu Kα diffractometer (German, Bruker Nano GmbH, Berlin, Germany, APLX-DUO, 40 kV, 40 mA) from 2° to 40° with a wavelength of 0.71 nm at a scan speed of 2° min^−1^. Raman spectroscopy (Thermo Fisher Scientific Company, Waltham, MA, USA). N_2_ adsorption–desorption measurements were carried out on a Micromeritics analyzer (ASAP 2460, Micromeritics Instrument Corp., Norcross, GA, USA). The final element contents of the prepared samples were determined through the Inductively Coupled Plasma Optical (ICP, */iCAP6300).

## 3. Results and Discussion

### 3.1. Morphology and Structure of Zn/NC, Co/NC, and Co-Zn/NC

**Figure 1:**
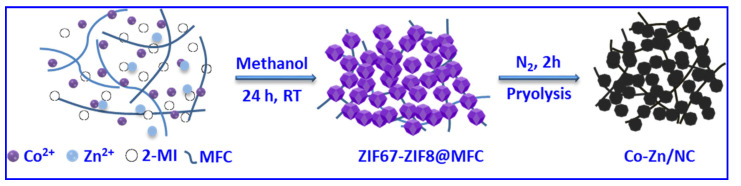
Process flowchart for the preparation of Co-Zn/NC.

**Figure 2:**
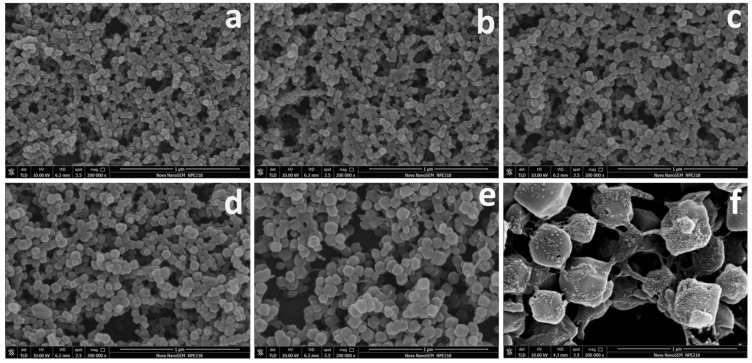
SEM images of ZIF8@MFC (**a**), ZIF67-ZIF8@MFC−10%, −20%, −30%, and −50% (**b**–**e**) and ZIF67@MFC (**f**).

The overall preparation procedure for Co-Zn/NC derived from ZIF67-ZIF8@MFC is illustrated schematically in Figure 1. ZIF67-ZIF8@MFC interconnected frameworks are prepared through the in situ growth of zeolitic imidazolate frameworks (ZIF67 and ZIF8) within MFC dispersed methanol solution, achieved by mixing MFC-dispersed methanol solution containing Zinc(II)/Co(II) nitrate with the methanol solution of 2-methylimidazole. Zn(II)/Co(II) ions adsorbed on MFC are complexed with 2-methylimidazole to form ZIF particles anchored on the MFC, thus resulting in the interconnected frameworks of ZIF particles connected by MFC and avoiding the agglomeration of ZIF particles and the aggregation of metal particles during calcination, so as to ensure abundant and evenly distributed active sites for efficient ORR. The SEM images of ZIF8@MFC, ZIF67-ZIF8@MFC−10%, −20%, −30%, and −50% and ZIF67@MFC with uniform size are shown in Figure 2. Both ZIF8 and ZIF67 crystals exhibit regular polyhedral morphology, but the crystal size of ZIF8 is significantly smaller compared to that of ZIF67. The ZIF67@ZIF8 synthesized under the coexistence of Co^2+^ and Zn^2+^ ions also exhibits dodecahedral morphology. In the ZIF@MFC series, as shown in Figure 2a–f, ZIF crystal size in ZIF@MFC samples gradually increases with an increasing Co content ranging from 0 to 100%. The corresponding sample sizes are as follows: ZIF8@MFC (50–60 nm), ZIF67-ZIF8@MFC−10% (55–65 nm), ZIF67-ZIF8@MFC−20% (65–70 nm), ZIF67-ZIF8@MFC−30% (80–90 nm), ZIF67-ZIF8@MFC−50% (100–110 nm), and ZIF67@MFC (~400 nm). The growth of ZIF crystals in MFC leads to the formation of interconnected three-dimensional structures where ZIF particle clusters are connected through MFC. This interconnection becomes more pronounced with increasing Co content. 

**Figure 3:**
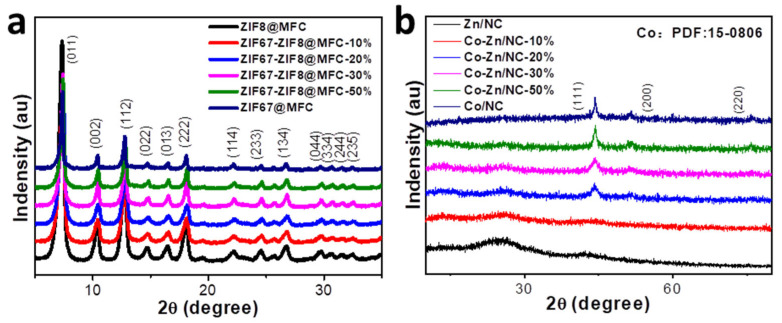
XRD patterns of ZIF8@MFC, ZIF67-ZIF8@MFC−10%, −20%, −30%, and −50%, and ZIF67@MFC (**a**) and Zn/NC, Co-Zn/NC−10%, −20%, −30%, and −50% and Co/NC (**b**).

The XRD patterns in Figure 3a show that the diffraction peaks of ZIF67@MFC, ZIF8@MFC, and ZIF67-ZIF8@MFC are well matched with those reported for ZIF67 and ZIF8 in previous literature [36], which proves the successful synthesis of ZIF67 and ZIF8 even in the presence of MFC. After calcination at 800 °C, the XRD patterns for corresponding resultants including Zn/NC, Co-Zn/NC-X (X = 10%, 20%, 30%, and 50%), and Co/NC are shown in Figure 3b. The diffraction peaks centered around 44.5°, 51.7°, and 76.1° belong to the (111), (200), and (220) crystal planes of face-centered cubic Co (PDF 015-0806) [37], respectively. The two wide diffraction peaks at 29° and 43° can be indexed to the (002) and (100) graphitic carbon planes of carbon. [38] The diffraction peak of Zn is not observable due to its relatively low content. 

**Figure 4:**
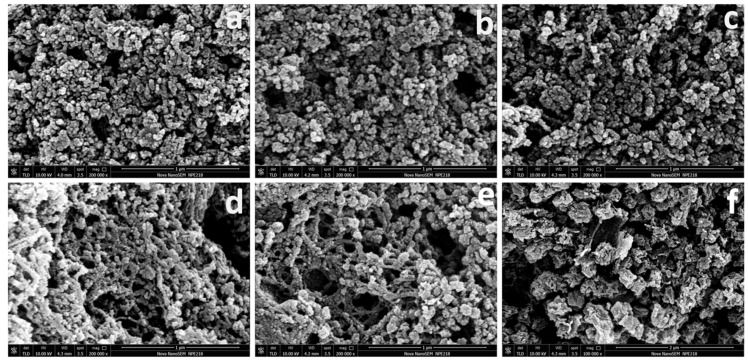
SEM images of Zn/NC (**a**), Co-Zn/NC−10%, −20%, −30%, and −50% (**b**–**e**), and Co/NC (**f**).

SEM images of Zn/NC, Co-Zn/NC-X (X = 10%, 20%, 30%, 50%), and Co/NC obtained by pyrolysis of ZIF8@MFC, ZIF67-ZIF8@MFC−10%, −20%, −30%, and −50%, and ZIF67@MFC at 800 °C are represented in Figure 4. The Zn/NC and Co-Zn/NC−10% and −20% synthesized with lower Co content exhibit a higher tendency to agglomerate during calcination due to their smaller ZIF particle sizes (Figure 4a–c). Notably, Co-Zn/NC−30% and −50% exhibit interconnected three-dimensional frame structures resembling fishing nets when the proportion of Co reaches 30% or higher. The larger size of ZIF particles formed with high Co content is more easily supported and connected by MFC, thereby mitigating agglomeration (Figure 4d,e). Although Co/NC (derived from ZIF67@MFC with a Co content of 100%) possesses ZIF particles large enough to avoid agglomeration under the support of MFC (Figure 4f), the high Co content (40.06%, Table 1) and absence of the macroporous structure found in ZIF8 (Figure S1) limit its suitability. 

The elemental contents of Zn/NC, Co-Zn/NC−10%, −20%, −30%, and −50%, and Co/NC determined by the ICP test are listed in Table 1. The Co content in the Zn/NC, Co-Zn/NC−10%, −20%, −30%, and −50%, and Co/NC samples increases with the increasing proportion of original Co, while the Zn content decreases significantly, which is notably lower than the theoretical Zn content. This is because the boiling point of Zn is 907 °C, a large amount of Zn has volatilized during the calcination process at 800 °C, and when the proportion of original Co reaches 30% or higher, the residual Zn after calcination has been reduced to almost negligible (just 0.70% for Co-Zn@NC−30%).

The BET-specific surface areas (SSAs) and pore-size distributions of ZIF8@MFC, ZIF67-ZIF8@MFC-X (X = 10%, 20%, 30%, 50%), ZIF67@MFC, ZIF8, and ZIF67@ZIF8 are measured via N_2_ adsorption-desorption experiments (Figure S1). As displayed in Figure S1a, the adsorption isotherm of ZIF67@MFC exhibits type Ⅰ isotherm characteristics with a microporous structure [39]. In contrast, ZIF8@MFC and ZIF67-ZIF8@MFC-X display a combination of type-I and type-II isotherms, indicating the coexistence of abundant microporous and macroporous structures [40]. The abrupt rise observed in type II isotherms at high relative pressures corresponds to the presence of macroporous structures [41]. The ZIF67-ZIF8@MFC−30% exhibits the highest specific surface area of 1043.76 m^2^/g among ZIF@MFC series samples, significantly surpassing 830.28 m^2^/g of ZIF67@MFC. Moreover, the pore size distributions are analyzed using the Barrett–Joyner–Halenda (BJH) model (Figure S1b,c) and the Horvath–Kawazoe (HK) model (Figure S1d). Except for that, there is no macropore structure for ZIF67@MFC (Figure S1b), whereas ZIF8@MFC and ZIF67-ZIF8@MFC-X all exhibit a hierarchical porous architecture consisting of micropores, hollow pores, and macropores, which is consistent with the isotherm results shown in Figure S1. The BET-specific surface areas and macroporous sizes of ZIF@MFC series samples are found to be smaller than those of ZIF8 and ZIF67@ZIF8, which can be attributed to the blocking caused by MFC during the preparation process in terms of penetration and support for the ZIF8 particles. The presence of micropores enhances the exposure of active sites, while the abundant macroporous structure facilitates the smooth diffusion of a large number of gases to the active site without retention, thereby ensuring overall catalytic efficiency [42]. Consequently, ZIF67-ZIF8@MFC−30% can be considered the optimal candidate due to its unique combination of ZIF67’s microporous structure and ZIF8’s macroporous structure, while simultaneously achieving a reduced cobalt content.

After calcination, the Raman spectra of the obtained samples of Zn/NC, Co-Zn/NC−10%, −20%, −30%, and −50%, and Co/NC are shown in Figure S2. The Raman spectra exhibit characteristic D and G peaks located at approximately 1345 and 1580 cm^−1^, respectively [43]. The G band is attributed to the E_2g_ vibrational mode of graphite, while the D band corresponds to the A_1g_ mode associated with disordered carbon structure and defects. The intensity ratio of D and G bands (I_D_/I_G_) is commonly employed to determine the degree of disorder of carbon materials [44]. The results demonstrated that the I_D_/I_G_ ratios of Zn/NC, Co-Zn/NC−10%, −20%, −30%, and −50%, and Co/NC were 1.51 > 1.39 > 1.33 > 1.24 > 1.12 > 0.98, respectively. With an increase in the proportion of Co content, there was a gradual reduction in defect sites within the carbon materials, leading to an enhancement in the degree of order and regularity in the carbon matrix as well as an increase in graphitization degree, indicating that higher metal content promotes catalytic graphitization.

**Figure 5:**
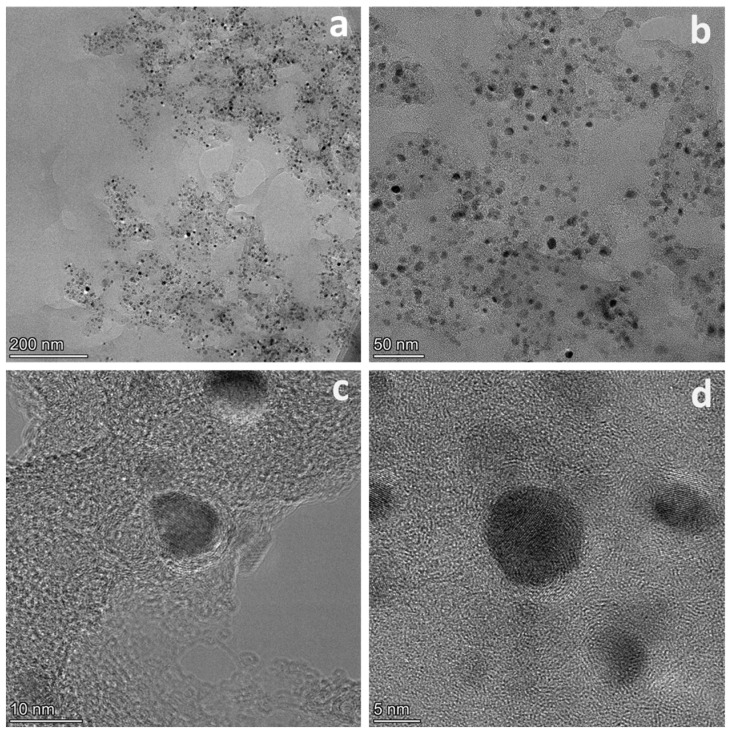
TEM (**a**,**b**) and HRTEM (**c**,**d**) of Co-Zn@NC−30%.

As shown in Figure 5a,b and Figure S3a, the TEM images of Co-Zn@NC−30% demonstrate that Co nanoparticles exhibit a uniform size distribution ranging from 5 to 10 nm and are well dispersed within the carbon matrix. The corresponding elemental mapping images and high-angle dark field scanning transmission electron microscopy (HAADF-STEM) of Co-Zn@NC−30% confirm the presence and uniform distribution of C, N, Co, and Zn elements (Figure S3b–f). In order to further investigate the effect of Co metal catalytic graphitization, HRTEM on Co-Zn@NC−30% was performed. The presence of imperfect graphitization layers surrounding Co nanoparticles and graphitization traces within the carbon matrix can be observed in Figure 5c,d. These graphitized carbons play a crucial role In enhancing catalyst stability.

### 3.2. Electrocatalytic Performance toward ORR of Zn/NC, Co/NC, and Co-Zn/NC Composites

**Figure 6:**
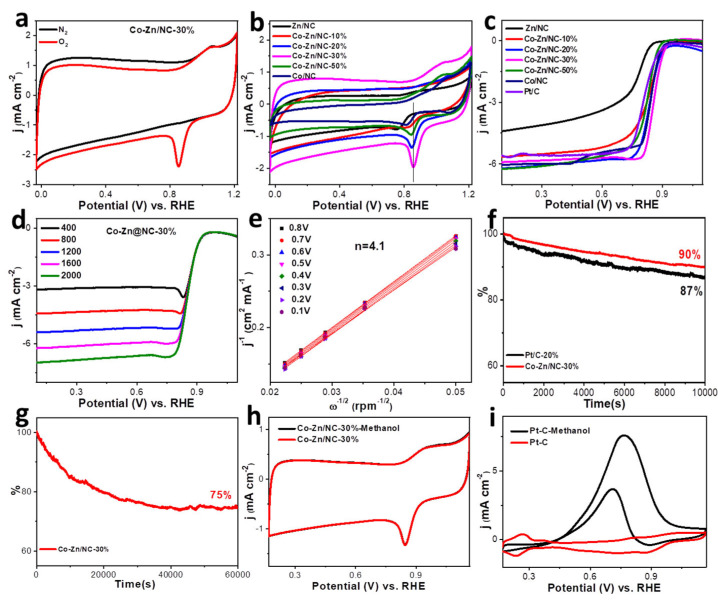
(**a**) CV curves of Co-Zn/NC−30% in N_2_/O_2_-saturated 0.1 M KOH with a scan rate of 10 mV s^−1^; (**b**) CV curves of Zn/NC, Co−Zn/NC−10%, −20%, −30%, and −50%, and Co/NC; (**c**) LSV curves of Zn/NC, Co−Zn/NC−10%, −20%, −30%, and −50% and Co/NC and commercial Pt/C 20% in O_2_-saturated 0.1 M KOH at a rotating rate of 1600 rpm; LSV curves (**d**) and K−L plots (**e**) of Co−Zn/NC−30%; (**f**) Current−time (i−t) chronoamperometric response of Co−Zn/NC−30% and Pt/C 20% in O_2_−saturated 0.1 M KOH at a rotating rate of 1600 rpm for 10,000 s; (**g**) Current-time (i-t) chronoamperometric response of Co−Zn/NC−30% in O_2_−saturated 0.1 M KOH at a rotating rate of 1600 rpm for 60,000 s; (**h**,**i**) CV curves of Co−Zn/NC−30% and Pt/C 20% with and without 3 wt.% CH_3_OH in 0.1 M KOH.

The electrocatalytic activities of Zn/NC, Co-Zn/NC−10%, −20%, −30%, and −50%, and Co/NC composites are first evaluated through CV curves in O_2_-saturated 0.1 M KOH at a scan rate of 10mV s^−1^ (Figure 6a,b). The oxygen reduction peak locations for these samples are presented in Table 2. The Co-Zn/NC−30% composite catalyst exhibits the most positive oxygen reduction peak at 0.857V vs. RHE. To further confirm the ORR catalytic activity of the prepared catalysts, LSV curves were performed using RDE at a scanning rate of 10 mV s^−1^ in O_2_-saturated 0.1 M KOH, and commercial Pt/C 20% was used for comparative analysis. As shown in Figure 6c and Table 2, the Co-Zn/NC−30% composite exhibits the optimal onset potential (E_0_) and half-wave potential (E_1/2_) of 0.95V vs. RHE and 0.825V vs. RHE, respectively, surpassing that of commercial Pt/C. These results unequivocally establish the excellent ORR electrocatalytic activity for Co-Zn/NC−30% composite, which should be attributed to its large active specific surface area derived from the hierarchical porous structure, as well as abundant active sites resulting from the uniform distribution of N and good dispersion of Co nanoparticles. 

RDE measurements are conducted with various rotation speeds ranging from 400 to 2000 rpm in O_2_-saturated 0.1 M KOH to obtain detailed information for the ORR mechanism of the Co-Zn/NC−30% composite. LSV curves display an increase in limiting current density as the rotation speeds increase (Figure 6d). The electron-transfer number (n) during the electrocatalytic reaction is calculated according to the Koutecky–Levich (K-L) equation based on the LSV curves at different rotations. The linearity of the K-L plots at different potentials indicated first-order reaction kinetics, which is related to the oxygen concentration. The n value for the Co-Zn/NC−30% composite is calculated to be 4.1, indicating an ideal four-electron transfer pathway toward the efficient ORR (Figure 6e).

Apart from their electrocatalytic activity, the electrochemical stability and resistance to methanol of ORR catalysts hold significant importance for their practical application. The stability of Co-Zn/NC−30% and Pt/C 20% is evaluated by Current-time (i-t) chronoamperometry in O_2_-saturated 0.1 M KOH at a rotating rate of 1600 rpm for 10,000 s. As shown in Figure 6f, the Co-Zn/NC−30% composite catalyst exhibits excellent stability with 90% retention of initial current, comparable to 87% for commercial Pt/C. After a long-term durability test for 60,000 s, the current retention rate can still be maintained at 75% (Figure 6g). Therefore, it is evident that the encapsulation effect of the graphite layer surrounding the Co nanoparticles significantly contributes to enhancing the stability of the Co-Zn/NC−30% composite catalyst. Moreover, as depicted in Figure 6h,i, the CV curve and oxygen reduction peak location of Co-Zn/NC−30% remain virtually unchanged before and after methanol addition. In contrast, the CV curve of Pt/C exhibited distinct methanol redox characteristics associated with methanol, thereby indicating excellent methanol tolerance of the Co-Zn/NC−30% composite catalyst. Additionally, as shown in Figure S4, the CV and LSV curves, as well as the i-t chronoamperometric response of the Co-Zn@NC−30% composite catalyst tested in O_2_-saturated 0.5 M H_2_SO_4_ indicate its ORR activity and good stability in acidic media. Considering that cobalt must be dissolved in a sulfuric acid solution, the efficient activity sites in acidic media should be porous N-doped carbon. Therefore, the Co-Zn/NC−30% composite catalyst is an excellent candidate catalyst for ORR due to its efficient activity and long-term stability in alkaline media, as well as its potential use as an ORR catalyst in acidic media.

## 4. Summary

In summary, Co-Zn@NC interconnected frameworks with uniformly dispersed Co nanoparticles and graphitic carbon are successfully synthesized via the in situ growth of ZIF67 and ZIF8 along with MFC, followed by pyrolysis. The Co-Zn/NC composite is prepared by combining Co-Zn/NC with perfluorosulfonic acid polymer. The Co-Zn@NC−30% composite catalyst exhibited excellent catalytic activity for ORR and good long-term durability surpassing that of commercial Pt/C in alkaline media, as well as potential applications in acidic media. The hierarchical porous structure coupled with the uniform distribution of Co nanoparticles and nitrogen doping contributed to the remarkable electrocatalytic activity, while the interconnected frameworks and graphitic carbon induce good stability. This strategy offers new insight for developing highly efficient and stable ORR catalysts. 

## Figures and Tables

**Table 1:** Elemental contents of Zn/NC, Co-Zn/NC−10%, −20%, −30%, −50%, and Co/NC tested by ICP.

Sample	Co (wt. %)	Zn (wt. %)
1-Zn@NC	0.03	14.68
2-Co-Zn@NC−10%	6.37	4.52
3-Co-Zn@NC−20%	13.03	2.59
4-Co-Zn@NC−30%	17.64	0.70
5-Co-Zn@NC−50%	25.34	0.33
6-Co@NC	40.06	0.041

**Table 2:** The ORR activity comparison of different composite catalysts.

Sample	CV (EORR vs. RHE)	E0 (V vs. RHE)	E1/2 (V vs. RHE)
1-Zn@NC	0.746	0.891	0.783
2-Co-Zn@NC−10%	0.791	0.953	0.830
3-Co-Zn@NC−20%	0.848	0.959	0.851
4-Co-Zn@NC−30%	0.857	0.974	0.858
5-Co-Zn@NC−50%	0.843	0.946	0.840
6-Co@NC	0.831	0.952	0.852
7-Pt/C−20%	-	0.950	0.825

## Data Availability

Data are contained within the article.

## Supplementary Materials

The following supporting information can be downloaded at: https://www.mdpi.com/article/10.3390/polym16040505/s1, Figure S1: N2 adsorption/desorption isotherms (**a**), BJH pore size distributions (**b**,**c**) and HK pore size distributions (**d**) of ZIF8@MFC, ZIF67-ZIF8@MFC−10%,−20%,−30%,−50%, ZIF67@MFC, ZIF8 and ZIF67@ZIF8; Figure S2: Raman spectra of Zn/NC, Co-Zn/NC−10%,−20%,−30%,−50% and Co/NC; Figure S3: TEM (**a**), the corresponding elemental mapping images (**b**–**e**) and HAADF-STEM (**f**) of Co-Zn@NC−30%; Figure S4: (**a**) CV curves of Co-Zn@NC−30% in N2/O2-saturated 0.5 M H2SO4 with a scan rate of 10 mV s^−1^; LSV curves (**b**) and Koutecky-Levich (K-L) plots (**c**) of Co-Zn@NC−30% in O2-saturated 0.5 M H2SO4 with a scan rate of 10 mV s^−1^; (**d**) I-t chronoamperometric response of Co-Zn@NC−30% and Pt/C 20% with a rotation speed of 1600 rpm in O2-saturated 0.5 M H2SO4 for 10000s; Table S1: Raw materials of ZIF8@MFC, ZIF67@MFC and ZIF67-ZIF8@MFC-X (X = 10%, 20%, 30%, 50%).
