# Supplementary material for: ZIF67-ZIF8@MFC-Derived Co-Zn/NC Interconnected Frameworks Combined with Perfluorosulfonic Acid Polymer as a Highly Efficient and Stable Composite Electrocatalyst for Oxygen Reduction Reactions
Source: Polymers (Basel). 2024 Feb 12;16(4):505. doi: 10.3390/polym16040505 (PMC10893250; doi:10.3390/polym16040505)
Supplement: Supplementary file 1 [file polymers-16-00505-s001.zip › polymers-2856118-supplementary.pdf]

Supporting information

ZIF67-ZIF8@MFC Derived Co-Zn/NC Interconnected Frameworks Combined with Perfluorosulfonic Acid Polymer as Highly Efficient and Stable Composite Electrocatalyst for Oxygen Reduction Reactions

Hongjie Meng[a], *Jingnan Song*[a*], *Yongming Zhang*[a*]

a School of Chemistry and Chemical Engineering, Center of Hydrogen Science, Shanghai Jiao Tong University, Shanghai 200240 (P. R. China)

*Corresponding author: Jingnan Song, Email: sjn199319@sjtu.edu.cn;

Yongming Zhang, Email: ymzhang@sjtu.edu.cn

Electrochemical characterization

All the electrochemical performances were examined by an Autolab PGSTAT302 (Metrohm, Netherland) electrochemical workstation. The catalyst ink was prepared by dispersing 1 mg catalyst into the mixed solution of 10 μL perfluorosulfonic acid polymer solution (5 wt.% Nafion 117 solution) and 200 μL hydroalcoholic solution (V_{water}: $V_{alcohol}$ = 4:1) under ultrasonic homogenization. All the working electrodes were prepared by drip-coating the above ink on the glassy carbon rotating disk electrode with a catalyst loading about 0.3 mg/cm^2. Cyclic voltammetry (CV) and linear sweep

voltammetry (LSV) curves were performed in 0.1 M KOH solution or 0.5 M H_2SO_4 solution with a scan speed of 10 mV s^{-1}. Before carrying on each ORR measurement, the electrolyte was bubbled with N_2 or O_2 for at least half an hour to build an N_2-saturated or O_2-saturated testing environment.

LSV curves were recorded with rotating rates from 400 to 2000 rpm by rotating disk electrode (RDE) cathodically. The durability was measured via current-time (i-t) chronoamperometric at 0.7V (vs. RHE) in O_2-saturated electrolyte. The electron transfer number (n) per O_2 during the ORR catalytic process can be calculated according to the RDE data and the Koutecky-Levich (K-L) equations as follows:

$$\frac{1}{J} = \frac{1}{J_L} + \frac{1}{J_K} = \frac{1}{B\omega^{1/2}} + \frac{1}{J_K} \quad (1)$$

$$B = 0.62nFD_{O_2}^{2/3}C_{O_2}^{b}v^{-1/6} \quad (2)$$

$$|J_k| = nFk_fC_{O_2}^{b} \quad (3)$$

Where J is the overall current density from practical measurements. J_K is the kinetic current, J_L is diffusion-limited current densities, ω represents the rotation rate (rad s^{-1}), F is the Faraday constant (96485 C mol^{-1}), n is transferred electron number, $D_{O_2}^{2/3}$ is the diffusion coefficient of O_2 (1.9×10^{-5}), $C_{O_2}^{b}$ is the bulk concentration of O_2 (1.2×10^{-3} mol cm^{-3}), v is kinetic viscosity (0.01 cm^2 s^{-1}), and k_f is the electron-transfer rate constant. Moreover, rotating ring-disk electrode (RRDE) of the catalyst was performed at 1600 rpm in 0.1 M KOH medium, the value of n and H_2O_2 yield could be determined according the following equations:

$$H_2O_2\% = 100 \times \frac{2I_r / N}{I_d + I_r / N} \quad (4)$$

$$n = \frac{4I_d}{I_d + I_r / N} \quad (5)$$

Where I_d and I_r are disk current and ring current, respectively, N is the H_2O_2 collection efficiency at the ring (N = 0.39).

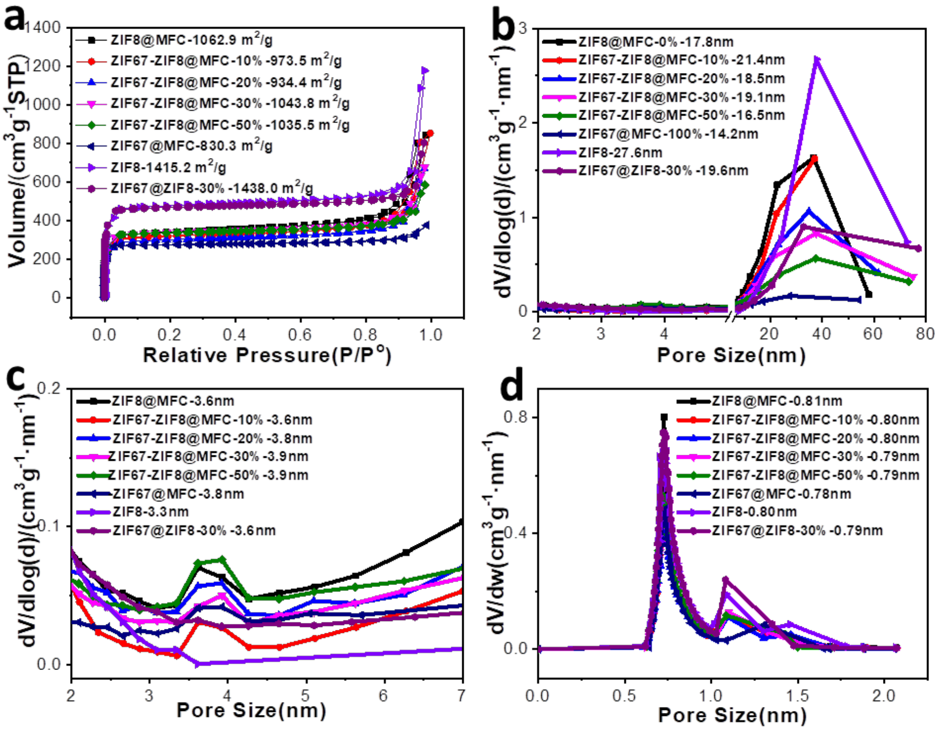

Figure S1. N_2 adsorption/desorption isotherms (a), BJH pore size distributions (b & c) and HK pore size distributions (d) of ZIF8@MFC, ZIF67-ZIF8@MFC-10%,-20%,-30%,-50%, ZIF67@MFC, ZIF8 and ZIF67@ZIF8.

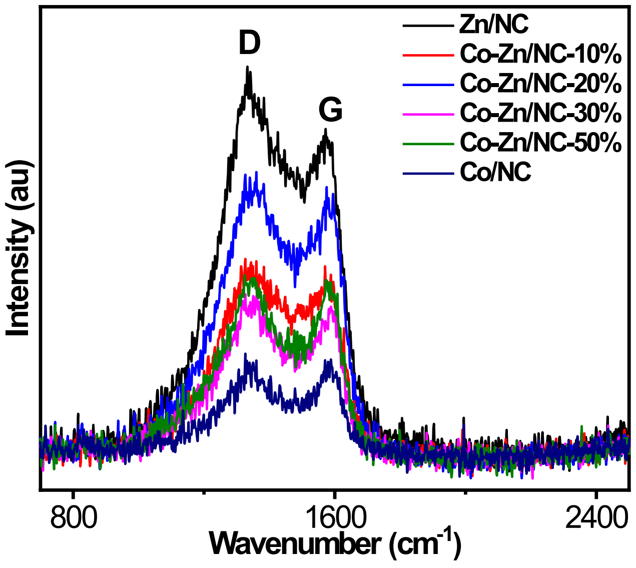

Figure S2. Raman spectra of Zn/NC, Co-Zn/NC-10%,-20%,-30%,-50% and Co/NC.

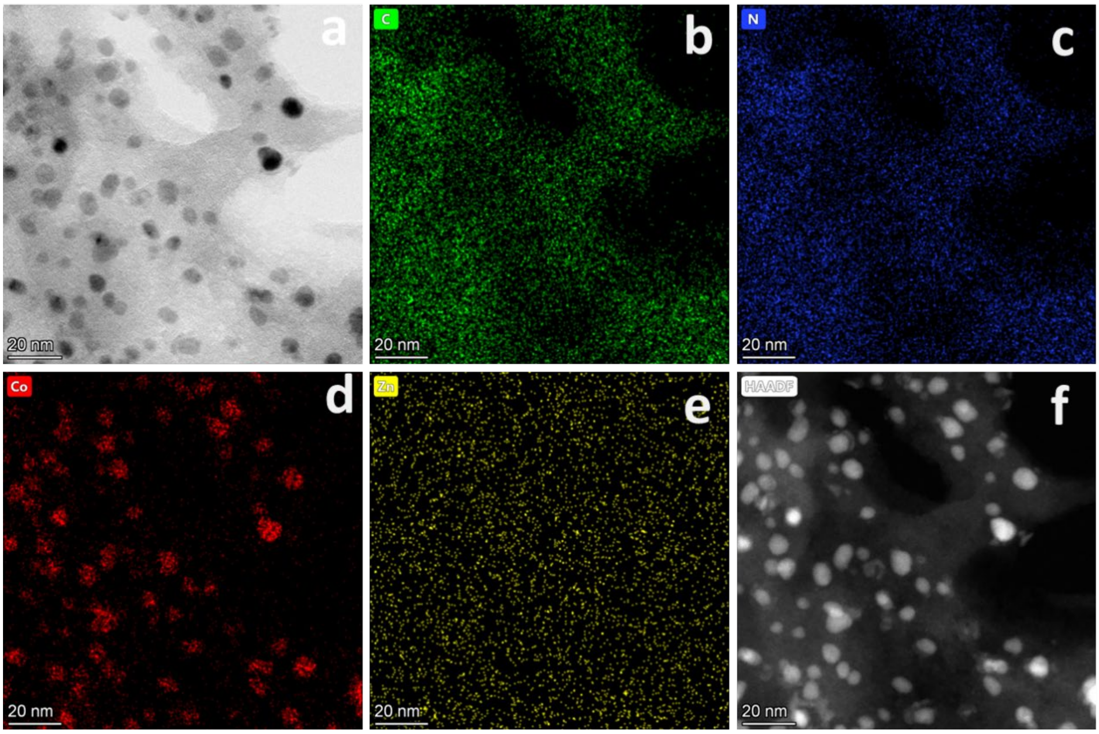

Figure S3. TEM (a), the corresponding elemental mapping images (b, c d & e) and HAADF-STEM (f) of Co-Zn@NC-30%.

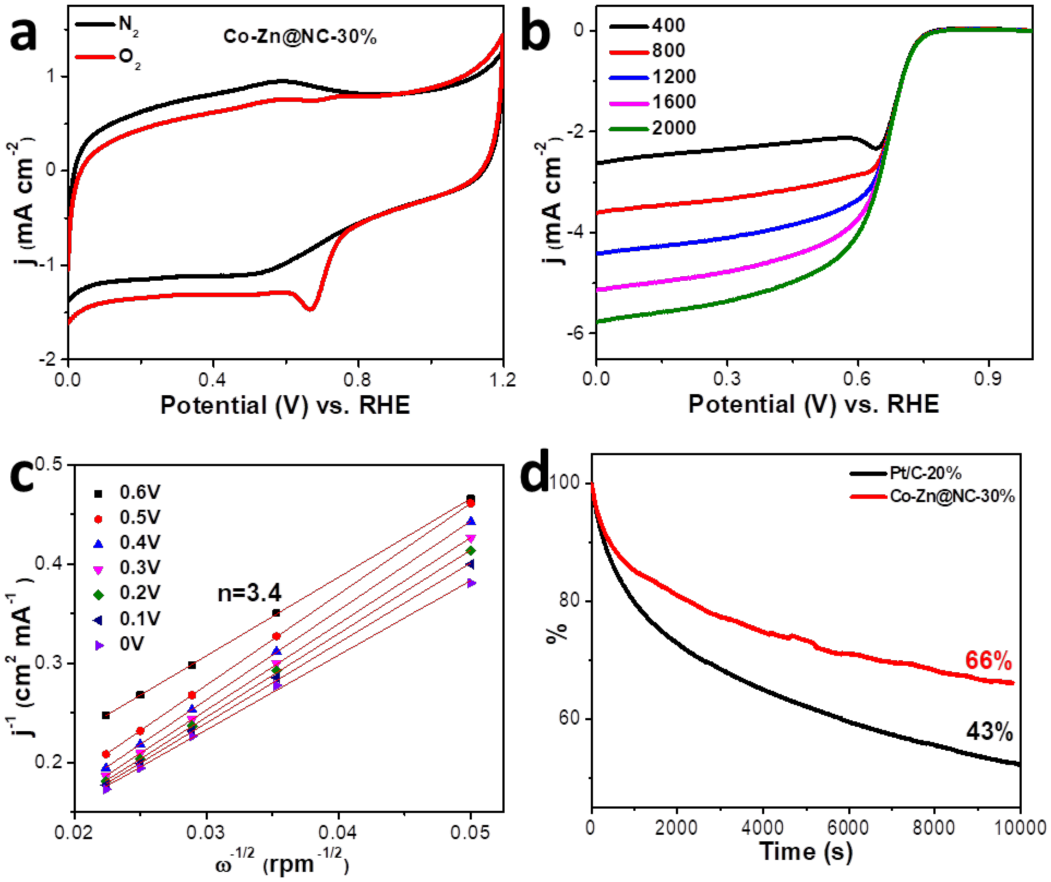

Figure S4. (a) CV curves of Co-Zn@NC-30% in N_2/O_2-saturated 0.5 M H_2SO_4 with a scan rate of 10 mV s^{-1}; LSV curves (b) and Koutecky-Levich (K-L) plots (c) of Co-Zn@NC-30% in O_2-saturated 0.5 M H_2SO_4 with a scan rate of 10 mV s-1; (d) I-t chronoamperometric response of Co-Zn@NC-30% and Pt/C 20% with a rotation speed of 1600 rpm in O_2-saturated 0.5 M H_2SO_4 for 10000s.

Table S1 Raw materials of ZIF8@MFC, ZIF67@MFC and ZIF67-ZIF8@MFC-X (X=10%, 20%, 30%, 50%)

Sample	$Co(NO_3)_2·6H_2O$/g	$Zn(NO_3)_2·6H_2O$/g	2-MI/g	MFC/g
**1-ZIF8@MFC**	0	0.730	1.630	1.000
**2-ZIF67-ZIF8@MFC-10%**	0.714 * 0.1=0.071	0.73 * 0.9=0.657	1.630	1.000
**3-ZIF67-ZIF8@MFC-20%**	0.714 * 0.2=0.143	0.73 * 0.8=0.584	1.630	1.000
**4-ZIF67-ZIF8@MFC-30%**	0.714 * 0.3=0.214	0.73 * 0.7=0.511	1.630	1.000
**5-ZIF67-ZIF8@MFC-50%**	0.714 * 0.5=0.357	0.73 * 0.5=0.365	1.630	1.000
**6-ZIF67@MFC**	0.714	0	1.630	1.000
